# Drug prescription for patients treated by dialysis: navigation in the fog

**DOI:** 10.1093/ckj/sfag134

**Published:** 2026-05-04

**Authors:** Christian Combe, Rukshana Shroff, Mandy Wan, Björn Meijers

**Affiliations:** U1219, Bordeaux Public Health, INSERM, Université de Bordeaux, Bordeaux, France; Department of Nephrology and Nutrition, Hospices Civils de Lyon, Centre Hospitalier Lyon-Sud, Pierre-Bénite, France; Centre Hospitalier d’Ardèche Méridionale, Aubenas, France; University College London Great Ormond Street Hospital and Institute of Child Health, London, UK; Pharmacy Department, Evelina London Children’s Hospital, Guy’s and St Thomas’ NHS Foundation Trust,, London, UK; Institute of Pharmaceutical Science, King’s College London, London, UK; Laboratory of Nephrology and Renal Transplantation, Department of Microbiology, Immunology and Transplantation, KU Leuven, Belgium; Nephrology Unit, UZ Leuven, Belgium

In an important paper in this issue of *Clinical Kidney Journal*, Touzot *et al*. [1] highlight a lack of evidence-based guidelines for drug prescribing for patients undergoing maintenance dialysis, emphasizing that nephrologists bear the primary responsibility for prescribing many classes of medications. Notably, the latest recommendations for dialysis date back to 2015 [2], and none of the recent KDIGO- Kidney Disease Improving Global Outcomes guidelines address drug prescribing for patients on dialysis. For instance, the KDIGO 2024 Clinical Practice Guideline for the Evaluation and Management of Chronic Kidney Disease [3] mentions ‘People receiving dialysis … are not the focus of this guideline’. In their review, Touzot *et al*. advocate for the use of several classes of drugs that have been shown to be effective in clinical trials that excluded patients on dialysis. While we concur with most of their proposals, it is essential to recognize that these recommendations largely stem from the authors’ clinical experience, expert opinion, and epidemiological data.

As members of the EuDial working group of the European Renal Association (ERA), we frequently encounter the evidence gap in drug studies relevant to dialysis, as noted by Touzot *et al*. This editorial aims to broaden the discussion around medications for people undergoing chronic dialysis.

A primary factor limiting pharmaceutical companies’ interest in dialysis studies is that the number of individuals requiring kidney replacement therapy (KRT) is rather low. In Europe, the prevalence of KRT in 2022 was 1074 patients per million population, with 56% of patients being treated by haemodialysis and 5% by peritoneal dialysis [4]. Thus, the number of individuals being treated by dialysis fulfills the definition of a rare disease [5] and peritoneal dialysis could be considered a very rare treatment. The situation is even more concerning for paediatric patients, where fewer than 3000 children aged 14 or younger were receiving KRT across 35 countries in the European Society of Paediatric Nephrology -ESPN /ERA Registry: all modalities of KRT for children thus represent a very rare condition in Europe [6]. With limited pharmaceutical investment, numerous studies in the field of dialysis are typically funded by nonprofit or governmental organizations [7–9].

A second important point about clinical trials carried out in patients treated with chronic dialysis is the lack of standardization in the technical aspects of the dialysis technique. For instance, in the most recent published trials in the field, patients ‘were receiving haemodialysis three or four times per week’ [8], or ‘at least three times per week’ [7], or ‘had been receiving maintenance dialysis for at least 3 months (haemodialysis, haemodiafiltration, or peritoneal dialysis)’ [9]. In essence, dialysis is the main factor determining the survival of patients who are dependent on it; in an analysis of nearly 16 000 patients from the DOPPS—Dialysis Outcomes and Patterns Study, first-year mortality was linked to catheter use, lack of predialysis care, and variables reflecting nutritional status [10], factors with a low likelihood of being influenced by medications, irrespective of their pharmacodynamic properties. Dialysis technique itself also influences the outcome of patients, for instance dialyser membrane type [11], or haemodiafiltration compared to haemodialysis [12, 13]. A further important consideration is that mortality rates in patients on dialysis vary considerably between countries with different income levels [14], complicating the interpretation of clinical outcomes.

A third point is that patients seen by nephrologists have the highest mean number of comorbidities, the highest mean number of prescribed medications, and the highest rate of death [15]. This may apply to patients treated by dialysis who accumulate the highest burden of comorbidities linked to chronic kidney disease. In the presence of such complex multimorbidity, the capacity of a single pharmacological intervention to independently improve clinical prognosis is markedly diminished compared to patients with fewer concurrent pathologies. For example, it is well established that phosphate accumulation in dialysis patients is a factor of cardiovascular morbidity and mortality. However, clinical trials conducted with various phosphate binders have not demonstrated a significant effect on major cardiovascular events despite lowering of serum phosphate levels [16]. An additional level of complexity is that the limited effectiveness of statin therapy in dialysis patients could be compromised by the presence of hyperphosphataemia [17]. Thus, in people treated by dialysis, cardiovascular risk factors are multiple, traditional or nontraditional, and intricately intertwined, which certainly explains in large part the lack of effectiveness of drug therapies in this population.

The management of children treated with dialysis presents additional complexity, as off-label drug use is widespread in paediatric care even in children with CKD who are not on dialysis. Clinical decision-making is therefore often informed by expert recommendations or extrapolation from studies in adults. Beyond the influence of CKD and dialysis modality, the need to account for developmental changes in organ maturation further complicates direct extrapolation.

The authors hope that what are seen as ‘contraindications’ due to KRT evolve into opportunities to extend drugs’ indications [1]; this is probably overly optimistic, but we agree that we need to make progress in the field of drug prescribing for patients treated by dialysis, a responsibility that currently resides largely with the prescribing nephrologist, in the absence of formal recommendations. Here, we offer some suggestions that we hope will be useful in this area:

- Manufacturers of certain new drugs could be required to conduct specific studies in patients treated by dialysis as a condition of approval, similar to the requirements mandated for paediatric populations by regulatory agencies in Europe and the USA [18]. What types of medicines might be subjected to such regulations? From our perspective, medications targeting the cardiovascular system in general, and medications affecting a significant proportion of patients undergoing dialysis, particularly diabetic patients, are of particular interest. Given the significant infection problems in dialysis, new antibiotics should also be included.
- As mentioned by Touzot *et al*. [1], and as discussed above, the dialysis technique itself might affect drug clearance and other pharmacokinetic parameters, in addition to the effect of dialysis *per se* on the patients’ outcomes. Thus, we believe that it is essential to standardize the dialysis technique in clinical drug trials, or at the very least to include subgroup analyses, for example by distinguishing between haemodialysis and peritoneal dialysis, or haemodialysis and haemodiafiltration.
- We strongly suggest that clinical trials select dialysis patients for inclusion as either incident or prevalent cases. Mortality is higher during the first months of dialysis, primarily due to factors that cannot be corrected by medication, such as morbidity related to vascular access, it decreases after the first year on dialysis [19]. Since dialysis vintage is a major factor influencing mortality and morbidity, it should be at least amongst the prespecified variables analysed in clinical studies.
- Given the vulnerability of patients treated with dialysis, and the potential risk of drug accumulation due to renal failure, it is important to detect adverse drug effects as early as possible; this could be one of the benefits of registries and databases. As mentioned by Touzot *et al*. [1], we may leverage the use of electronic health records, data warehouses, and artificial intelligence to detect adverse drug effects, especially since dialysis patients are seen very frequently (156 times a year) in dialysis care facilities.
- Recruiting patients undergoing dialysis who are on multiple medications into clinical trials presents several challenges. Target emulation trials [20] might give answers to clinically important questions, offering a level of evidence that, although not equivalent to that of traditional randomized controlled trials, may provide useful information.
- Patients’ organizations should be included in the design of clinical trials, with an emphasis on Patients Reported Outcomes Measures, as quality of life in dialysis is amongst the lowest in patients with chronic illnesses.

Touzot *et al*. [1] should be heralded for asking the right questions regarding the prescription of dialysis medications and for their valuable suggestions, which we fully endorse. We believe that our additional suggestions are feasible, including in terms of regulatory constraints and cost. Given the relatively low number of patients treated by dialysis, clinical trials in the field will need public funding. This will require an investment from nephrologists and patients to raise awareness among health authorities, particularly at the European level, of the burden of chronic kidney disease at the replacement stage. The patients we serve truly deserve access to the best treatments, with a rigorous assessment tailored to their dialysis treatment context.
